# Effect of backscatter radiation on the occupational eye-lens dose

**DOI:** 10.1093/jrr/rrae034

**Published:** 2024-05-31

**Authors:** Saya Ohno, Satoe Konta, Ryota Shindo, Keisuke Yamamoto, Rio Isobe, Yohei Inaba, Masatoshi Suzuki, Masayuki Zuguchi, Koichi Chida

**Affiliations:** Department of Radiological Technology, Tohoku University Graduate School of Medicine, 2-1 Seiryo, Aoba, Sendai 980-8575, Japan; Department of Radiological Technology, Tohoku University Graduate School of Medicine, 2-1 Seiryo, Aoba, Sendai 980-8575, Japan; Department of Radiological Technology, Tohoku University Graduate School of Medicine, 2-1 Seiryo, Aoba, Sendai 980-8575, Japan; Department of Radiological Technology, Tohoku University Graduate School of Medicine, 2-1 Seiryo, Aoba, Sendai 980-8575, Japan; Department of Radiological Technology, Tohoku University Graduate School of Medicine, 2-1 Seiryo, Aoba, Sendai 980-8575, Japan; Department of Radiological Technology, Tohoku University Graduate School of Medicine, 2-1 Seiryo, Aoba, Sendai 980-8575, Japan; Division of Radiological Disasters and Medical Science, Department of Disaster Medicine, International Research Institute of Disaster Science, Tohoku University, 6-6-4, Aoba, Sendai 980-8579, Japan; Department of Radiological Technology, Tohoku University Graduate School of Medicine, 2-1 Seiryo, Aoba, Sendai 980-8575, Japan; Division of Radiological Disasters and Medical Science, Department of Disaster Medicine, International Research Institute of Disaster Science, Tohoku University, 6-6-4, Aoba, Sendai 980-8579, Japan; Department of Radiological Technology, Tohoku University Graduate School of Medicine, 2-1 Seiryo, Aoba, Sendai 980-8575, Japan; Department of Radiological Technology, Tohoku University Graduate School of Medicine, 2-1 Seiryo, Aoba, Sendai 980-8575, Japan; Division of Radiological Disasters and Medical Science, Department of Disaster Medicine, International Research Institute of Disaster Science, Tohoku University, 6-6-4, Aoba, Sendai 980-8579, Japan

**Keywords:** eye lens dose, radiation measurement, radiation safety, interventional radiology (IVR), fluoroscopically guided procedures, 3-mm dose-equivalent [Hp(3)], Pb glasses (lead eyewear), radiation protection

## Abstract

We quantified the level of backscatter radiation generated from physicians’ heads using a phantom. We also evaluated the shielding rate of the protective eyewear and optimal placement of the eye-dedicated dosimeter (skin surface or behind the Pb-eyewear). We performed diagnostic X-rays of two head phantoms: Styrofoam (negligible backscatter radiation) and anthropomorphic (included backscatter radiation). Radiophotoluminescence glass dosimeters were used to measure the eye-lens dose, with or without 0.07-mm Pb-equivalent protective eyewear. We used tube voltages of 50, 65 and 80 kV because the scattered radiation has a lower mean energy than the primary X-ray beam. The backscatter radiation accounted for 17.3–22.3% of the eye-lens dose, with the percentage increasing with increasing tube voltage. Furthermore, the shielding rate of the protective eyewear was overestimated, and the eye-lens dose was underestimated when the eye-dedicated dosimeter was placed behind the protective eyewear. We quantified the backscatter radiation generated from physicians’ heads. To account for the effect of backscatter radiation, an anthropomorphic, rather than Styrofoam, phantom should be used. Close contact of the dosimeter with the skin surface is essential for accurate evaluation of backscatter radiation from physician’s own heads. To assess the eye-lens dose accurately, the dosimeter should be placed near the eye. If the dosimeter is placed behind the lens of the protective eyewear, we recommend using a backscatter radiation calibration factor of 1.2–1.3.

## INTRODUCTION

It is essential to reduce patient and occupational exposure to radiation [1, 2]. Many studies have explored radiation dose optimization, and protection from occupational radiation exposure, particularly during interventional radiology (IR) [3–5].

IR procedures are increasingly being performed; this has occurred especially over the past several decades [6–8]. During IR procedures, patients and physicians are often exposed to high levels of radiation [9–11], leading to skin erythema and cataracts [12, 13]. In particular, IR physicians are exposed to scatter radiation from patients [14, 15]. The International Commission on Radiological Protection (ICRP) has reduced the occupational dose limit for the eye-lens to 20 mSv/year, averaged over 5 years, with no annual exposure exceeding 50 mSv [16]. This is a significant decrease from the previous dose limit of 150 mSv/year. Therefore, it is essential to evaluate and reduce the occupational radiation exposure to the eyes of IR physicians.

The eye-lens dose of IR physicians is produced by direct radiation to the eye and backscatter radiation generated from their own heads [17]. However, no previous studies have quantified the contribution of backscatter radiation. The eye-lens dose of IR physicians is often measured using dedicated direct eye dosimeters; these dosimeters can measure 3-mm dose equivalent, Hp(3) [18]. The most commonly used dosimeters include DOSIRIS™ and Vision®. Although these are thermoluminescent dosimeters, they have different placements. DOSIRIS™ is placed near the eye, whereas Vision® is placed behind the lens of the protective eyewear. A previous study demonstrated that the dose measured from behind the eyewear lens underestimated the eye-lens dose [19–21]. However, the study used Monte Carlo simulation, rather than using phantom or actual measurements.

IR physicians often use radiation protective eyewear to protect their eye lens, but conventional 0.75-mm Pb-equivalent eyewear is uncomfortable to wear during IR procedures, due to their heavy weight and poor fit [22, 23]. On the other hand, 0.07-mm Pb-equivalent eyewear is lightweight but has lower shielding rates due to low Pb-equivalent [18, 24]. Furthermore, air in the gap between the eyes and the protective eyewear can reduce the shielding rates and increase the eye lens exposure [25, 26]. Thus, the novel 0.07-mm Pb-equivalent protective eyewear was developed, based on 3D molded lenses to fit the user’s face. This novel eyewear can address several limitations of the conventional eyewear; however, few studies have evaluated the effectiveness of the novel eyewear [27].

 In this study, we quantified the contribution of backscatter radiation generated from the head to the eye-lens dose using a phantom. Then, we assessed the effect of different tube voltages on the backscatter radiation dose. Furthermore, we evaluated the effect of backscatter radiation on the shielding rate of the novel 0.07-mm Pb-equivalent protective eyewear. We also evaluated the optimal placement of the eye-dedicated dosimeter.

## MATERIALS AND METHODS

**Fig. 1:**
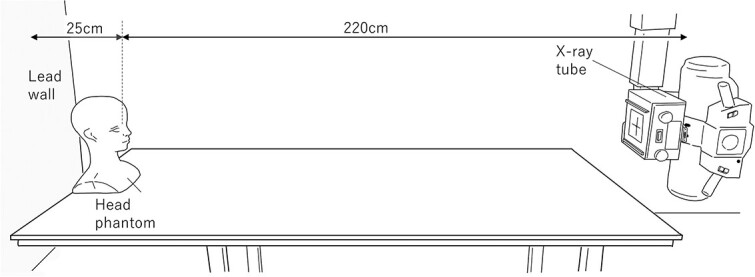
Illustration of the measurement geometry. The distances from the X-ray tube’s focus to the left eye of the phantom (Styrofoam or anthropomorphic) head and from the left eye of the phantom head to the lead wall were 220 and 25 cm, respectively.

We performed using diagnostic X-rays using tube voltages of 50, 65 and 80 kV based on the scatter radiation effective energy for fluoroscopy reported by Masterson *et al.* [28]. Masterson [28] reported that the scatter radiation effective energy range is in the 20–80 keV range; therefore, our X-rays (effective energy) are included in this range (Table 1). The radiological parameters were set at 1 mA, 60 s and fluoroscopic mode. We measured the half value layers of each tube voltage (Table 1). A 40 × 40-cm radiation field was set to ensure that the entire phantom was covered. The measurements are shown in Fig. 1. All measurements were conducted anteroposterior geometry to guarantee the repeatability of the measurement geometry by reference to a previous study [29]. The source and dosimeter distances were kept longer (220 cm) to provide a uniform distribution of the X-ray intensity. A radiophotoluminescence glass dosimeter (RPLD) with an Sn filter for low-energy compensation (GD-352 M), made of silver-activated phosphate glass, was placed on the phantom’s left eye at a distance of 25 cm from the lead wall (the influence of scatter radiation from the lead wall was ignored.). Although the RPLDs are direction-dependent [30], the direction could be ignored because the measurements were obtained in anteroposterior geometry. We used the Dose-Ace (FDG-1000) measurement/readout system.

**Table 1:** Half-value layers according to tube voltage

Tube Voltage (kV)	Half Value Layer (mmAl)	Effective Energy (keV)
50	2.0	28
65	2.5	31
80	3.2	34

RPLDs have excellent dose linearity, reproducibility and batch uniformity [31]. Compared with thermoluminescent dosimeters, RPLDs have almost no fading and can provide repeated readouts [30]. Before the RPLDs were used, they were annealed (400°C, 30 min) to reset the accumulated dose. After the measurements were obtained, the RPLDs were preheated to stabilize the luminescence before readout.

### Effect of the backscatter radiation on the eye-lens dose

**Fig. 2:**
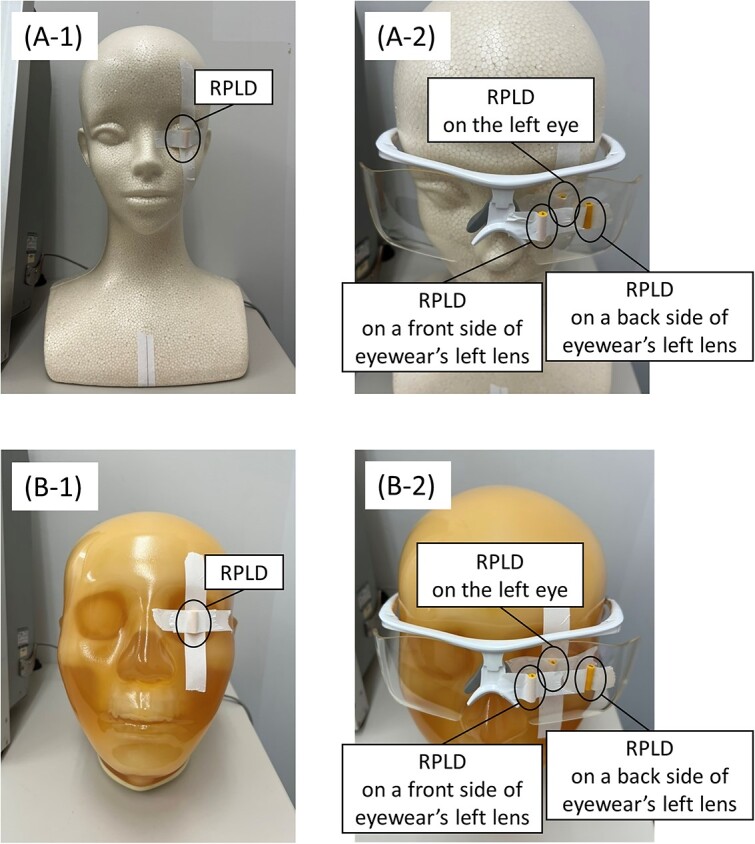
Photographs of Styrofoam (A) and anthropomorphic (B) phantom heads with and without the protective eyewear. In the phantom without the protective eyewear, a dosimeter was attached to the left eye (A-1, B-1). In the phantom with the protective eyewear, three dosimeters were attached: one on the phantom’s left eye and the others on the front and back sides of the left lens of the protective eyewear (A-2, B-2). Considering that Styrofoam absorbs minimal X-ray radiation, we assumed that the Styrofoam phantom had negligible backscatter radiation.

We quantified the effect of backscatter radiation on the eye-lens dose using two different types of head phantoms. The Styrofoam head phantom had negligible backscatter radiation from the head (Fig. 2; A-1; Dose_Styrofoam_), whereas the anthropomorphic head phantom had non-negligible backscatter radiation (Fig. 2; B-1; Dose_anthropomorphic_). An RPLD was placed on the surface of the phantom’s left eye, with a tube voltage of 50–80 kV. We calculated the backscatter radiation level (percentage) using the following equation (1):


(1) \begin{align*}& \mathrm{Backscatter}\ \mathrm{radiation}\ \mathrm{content}\ \left[\%\right]=\nonumber\\&\qquad\frac{{\mathrm{Dose}}_{\mathrm{Anthropomorphic}}-{\mathrm{Dose}}_{\mathrm{Styrofoam}}}{{\mathrm{Dose}}_{\mathrm{Anthropomorphic}}}\times 100 \end{align*}

### Effect of backscatter radiation on the protective eyewear

We evaluated the effects of backscatter radiation on the shielding rate of the protective eyewear. The novel 0.07-mm Pb-equivalent protective eyewear has 3D molded Pb-acrylic lenses that can attenuate the scatter radiation from below and the sides of the head. Furthermore, it is lightweight and can be adjusted at the nose pad and at the temples. These changes minimize the gap between the lenses and the face, thereby increasing the shielding rate.

#### Effect of backscatter radiation on the shielding rate

First, we evaluated the changes in the shielding rate due to backscatter radiation. We used two head phantoms and the novel 0.07-mm Pb-equivalent protective eyewear, with a tube voltage of 65 kV. Three RPLDs were used: one was placed on the surface of the phantom’s left eye (Dose_surface_) and the remaining were placed on front and behind the side of the eyewear (left lens, Fig. 2; A-2 and B-2; front-side measurements: Dose_front_, behind-side measurements: Dose_behind_). Three RPLDs were placed without overlap and were irradiated simultaneously for ignoring variations in the X-ray output. Then, we calculated the shielding rates behind the eyewear (2) and on the eye surface (3) using following equations:


(2) \begin{equation*} {\displaystyle \begin{array}{c}{\mathrm{Shielding}\ \mathrm{Rate}}_{\mathrm{behind}}\left[\%\right]=\frac{{\mathrm{Dose}}_{\mathrm{front}}-{\mathrm{Dose}}_{\mathrm{behind}}}{{\mathrm{Dose}}_{\mathrm{front}}}\times 100\end{array}} \end{equation*}

(3) \begin{equation*} {\displaystyle \begin{array}{c}{\mathrm{Shielding}\ \mathrm{Rate}}_{\mathrm{surface}}\left[\%\right]=\frac{{\mathrm{Dose}}_{\mathrm{front}}-{\mathrm{Dose}}_{\mathrm{surface}}}{{\mathrm{Dose}}_{\mathrm{front}}}\times 100.\end{array}} \end{equation*}

#### Effect of dosimeter placement on the shielding rate

Second, we evaluated the effects of dosimeter placement and tube voltage on the shielding rate. We used the anthropomorphic head phantom and protective eyewear with tube voltages of 50, 65 and 80 kV. Three RPLDs were placed in similar positions to those of the previous experiment (Fig. 2; A-2). We calculated the shielding rates with dosimeter behind the eyewear and on the eye surface using the aforementioned equations (2, 3).

### Statistical analysis

The results are presented as the mean and standard deviation of four measurements.

The Wilcoxon rank-sum test was used to compare the absorbed doses and shielding rates between the Styrofoam and anthropomorphic phantoms, as well as the shielding rates between dosimeters placed behind the eyewear and on the left eye surface. *P*-value <0.05 was considered to indicate statistical significance.

## RESULTS

### Effect of backscatter radiation on the eye-lens dose

**Fig. 3:**
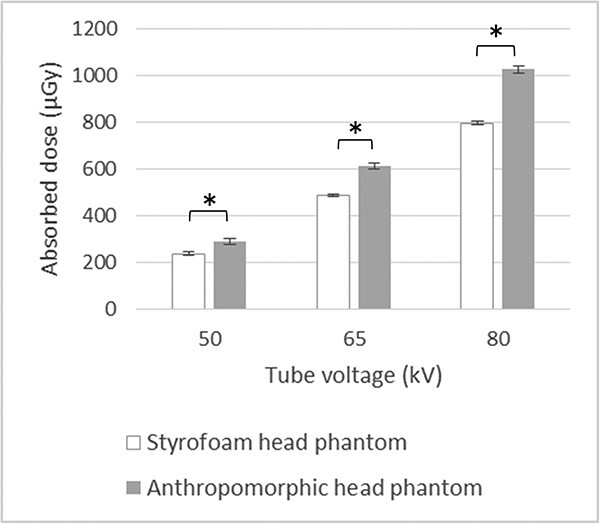
Doses at the left eye of the head phantom. Tube voltages of 50, 65 and 80 kV are shown. ^*^P < 0.05.

Figure 3 presents the radiation doses at the left eye of each phantom. At all tube voltages, doses were significantly higher in the anthropomorphic phantom than in the Styrofoam phantom. Table 2 summarizes the contribution of backscatter radiation to the eye-lens dose; this contribution increased with increasing tube voltage.

**Table 2:** Effect of backscatter radiation at each tube voltage. We calculated the proportion of eye-dose accounted for by the backscatter radiation in the anthropomorphic phantom

	50 kV	65 kV	80 kV
Styrofoam head phantom (μGy)	239.4 ± 6.0	489.8 ± 5.6	798.5 ± 8.3
Anthropomorphic head phantom (μGy)	289.6 ± 12.3	615.4 ± 14.3	1028.0 ± 14.0
Content of backscatter radiation (%)	17.3	20.4	22.3

### Effect of backscatter radiation on the protective eyewear

#### Effect of backscatter radiation on the shielding rate

**Fig. 4:**
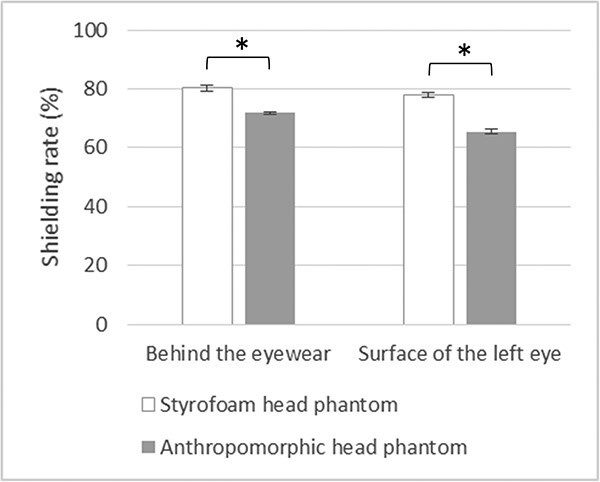
Shielding rates of the protective eyewear measured from behind the left lens of the protective eyewear and on the surface of the left eye. Tube voltage of 65 kV is shown. ^*^P < 0.05.

Figure 4 shows the shielding rates of the protective eyewear according to the dosimeter position. At both measurement positions, the shielding rates of the protective eyewear for the Styrofoam phantom were significantly higher than those for the anthropomorphic phantom. When the dosimeters were placed behind the eyewear, the shielding rate of the protective eyewear for the Styrofoam phantom was 11% higher than that for the anthropomorphic phantom. On the other hand, when the dosimeters were placed on the left eye surface, the shielding rate for the Styrofoam phantom was 20% higher than that for the anthropomorphic phantom. However, the shielding rates of the protective eyewear for the Styrofoam phantom were similar with both dosimeter positions.

#### Effect of the measurement position on the shielding rate

**Fig. 5:**
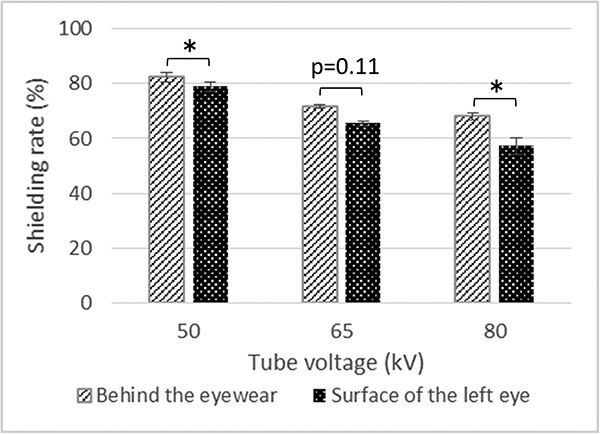
Shielding rates of the protective eyewear at each tube voltage measured behind the left lens of the protective eyewear and on the surface of the left eye of the anthropomorphic phantom. ^*^P < 0.05.

Figure 5 shows the shielding rates of the protective eyewear at each tube voltage for the anthropomorphic phantom. At 50 and 80 kV, the shielding rates of the protective eyewear were significantly higher behind the eyewear than on the left eye surface. At 65 kV, the shielding rate of the protective eyewear was higher behind the eyewear than on the left eye surface, although the difference was not statistically significant. We evaluated the degree of overestimation of the shielding rate of the protective eyewear when the dosimeter was placed behind the eyewear (Table 3). The degree of overestimation of the shielding rate of the protective eyewear increased with increasing tube voltage. As a result of the overestimation, a backscatter radiation calibration factor of 1.13–1.33 is required for the dose measured from behind the eyewear to match that obtained from the left eye surface (Table 4). The calibration factor also increased with increasing tube voltage.

**Table 3:** Relationship between the shielding rates measured behind the eyewear and on the eye surface of the anthropomorphic phantom. The upper two steps indicate the shielding rates at each position. The lowest step indicates the degree of overestimation of the shielding rate when the dosimeter was placed behind the eyewear

	50 kV	65 kV	80 kV
Shielding rate behind the eyewear (%)	82.5 ± 1.8	71.7 ± 0.6	68.1 ± 1.3
Shielding rate at surface of the eye (%)	78.9 ± 1.9	65.5 ± 0.8	57.4 ± 3.0
How much is overestimated the shielding rate (%)	5	10	19

**Table 4:** Relationship between the doses measured behind the eyewear and on the eye surface of the anthropomorphic phantom. The upper two steps indicate the doses measured at each position, and the lowest step indicates the calibration factor for measurements obtained from behind the eyewear

	50 kV	65 kV	80 kV
Dose behind the eyewear (μGy)	43.4 ± 2.7	128.2 ± 6.3	262.5 ± 23.4
Dose at surface of the eye (μGy)	52.3 ± 2.0	156.5 ± 3.8	349.2 ± 20.6
Calibration factor	1.20	1.22	1.33

## DISCUSSION

Given the increasing number of IR procedures, the medical radiation exposure should be reduced [32–38]. Multiple recent studies have evaluated the radiation exposure of patients and healthcare workers [39–45]. Reducing the patient radiation dose is important to reduce the healthcare worker dose (i.e. occupational exposure) [46–52]. Many studies have evaluated the medical radiation dose, including those performed at our institution [53–62].

The ICRP has reduced the occupational dose limit for eye-lens from 150 to 20 mSv/year; therefore, IR physicians should assess and reduce their eye doses.

The eye-lens dose should be measured accurately. Although IR physicians are exposed to scatter radiation mainly from the patient’s body and incidental backscatter radiation from IR physicians’ heads, previous studies have focused only on scatter radiation from patients. To our knowledge, no previous studies have quantified the backscatter radiation to the eye lens [20, 63]. Therefore, we quantified the backscatter radiation to the eye lens and demonstrated that the eye dose of IR physicians is contributed to by incident (scattered) radiation from patients as well as backscatter radiation from the physicians’ heads. We also assessed the effect of dosimeter position on the eye-lens dose and shielding rate of the protective eyewear.

In eye dosimetry, backscatter radiation from the head is not negligible. The backscatter radiation accounted for 17.3–22.3% of the eye-lens dose, and the proportion increased with increasing tube voltage. Therefore, if IR physicians protect their face and head, the amount of backscatter radiation generated would be reduced by 17.3–22.3%.

We also found that the shielding rate of the protective eyewear is overestimated if backscatter radiation from the physician’s own head is not evaluated. Though there is a phantom study that used Styrofoam to evaluate the efficiency of protective eyewear [29], this study suggests that when shielding rates are assessed in phantom studies, the backscatter radiation must be evaluated using an anthropomorphic phantom.

When measuring the eye-lens dose when protective eyewear is worn, dosimeters should be placed on skin surface near the eye because the backscatter radiation dose decreases with increasing distance from the skin surface.

The shielding rate of the protective eyewear is overestimated by 5–19% and the eye-lens dose is underestimated when the dosimeter is placed behind the lens of the protective eyewear. Certain eye-lens dosimeters, such as Vision®, are placed behind the lens of the protective eyewear. When such dosimeters are used, we recommend that the effect of backscatter radiation should be adjusted using a backscatter radiation calibration factor of 1.2–1.33.

In a phantom study, Domienik *et al.* [64] calculated a calibration factor of 1.6–2.0 when using two eyewear types with a Pb-equivalent of 0.75 mm, tube voltage of 80 kV and the dosimeter placed on the upper back of the lens (i.e. typical position of Vision®). On the other hand, Silva *et al.* [19] performed Monte-Carlo simulation and found that the eye surface dose was 1.2–2.5-fold higher than that under the glass lens when eyewear with a Pb-equivalent of 0.75 mm and a tube voltage of 90 kV were used.

In this study, we calculated a backscatter radiation calibration factor of 1.2–1.33, which is lower than that of previous studies. This is because the calibration factor calculated in the present study was only based on the backscatter radiation, whereas those calculated in previous studies were based on other sources of radiation in addition to the backscatter radiation (i.e. causes of air-gap between the eyes and protective eyewear). Therefore, to assess the eye-lens dose more accurately, the dosimeter should be placed as close to the eye as possible, rather than behind the lens of the protective eyewear.

In summary, the ICRP has recommended that the occupational eye-dose limit should be lowered to 20 mSv/year. The need to reduce the eye dose has led to significant interest in dosimetry and eye protection for IR physicians. The eye dose includes not only incident eye exposure but also backscatter radiation from the IR staff’s own head. However, the level of the contribution of backscatter radiation is unclear. In this study, the levels of backscatter radiation from IR physicians’ own heads were evaluated using a phantom. We investigated the optimal position of eye dosimeters (near the eye surface or behind the Pb eyewear) when Pb eyewear is used. Styrofoam (without backscatter) and anthropomorphic (with backscatter) phantoms were used for evaluation. Fluoroscopy-mode X-ray (tube voltages: 50, 65 and 80 kVp) were irradiated to the frontal side of the head phantom. Radiation doses were measured using RPLDs. We also measured the eye dose when Pb eyewear was used. The backscatter radiation accounted for 17.3–22.3% of the eye dose and the proportion increased with increasing tube voltage. Therefore, the contribution of backscatter radiation was lower when the eye dosimeter was placed behind the Pb eyewear, leading to the underestimation of the eye dose and overestimation of the shielding effect of Pb-eyewear. To determine the accurate eye dose, backscatter radiation from IR physicians’ own heads cannot be ignored. We quantified backscatter radiation from IR physicians’ own heads. The eye dosimeter should be placed near the eye surface for an accurate evaluation of eye-lens dose, including backscatter radiation dose. When the eye dosimeter was placed behind the Pb-eyewear, the eye dose, particularly the backscatter radiation component, was underestimated; therefore, we recommend using a backscatter radiation calibration factor of 1.2–1.33.

### Limitations

This study had several limitations. First, the actual level of backscatter radiation received by IR physicians may differ from our results because we conducted measurements while focusing on the repeatability of measurement geometry. Consequently, factors such as the distance between the radiation source and eyes, the irradiation direction and the scattering from the physician’s body may vary in daily practice.

Second, we irradiated the front of the phantom. In practice, IR physicians are often exposed from the lower left side because the X-ray tube is placed on the left side of physicians.

## CONCLUSION

There are two sources of eye radiation exposure: incident exposure and backscatter radiation from the physician’s own head. Backscatter radiation accounted for 17.3–22.3% of the eye-lens dose; this percentage increased with increasing tube voltage. Therefore, backscatter radiation from the physician’s own head accounts for a significant proportion of the eye-lens dose.

A Styrofoam phantom is associated with almost no backscatter radiation and leads to the overestimation of the shielding rate of the protective eyewear. Therefore, anthropomorphic phantoms should be used in phantom studies of backscatter radiation. Additionally, the dosimeter should be placed on the skin surface near the eye, rather than behind the lens of the eyewear. The eye dose was underestimated by dosimeters placed behind the lens of the eyewear because of markedly decreased backscatter radiation from the head. When the dosimeter is placed behind the lens of the eyewear with a Pb-equivalent of 0.07 mm, we recommend using a backscatter radiation calibration factor of 1.2–1.33, depending on the tube voltage.
